# Stability of gaseous volatile organic compounds contained in gas cylinders with different internal wall treatments

**DOI:** 10.1525/elementa.366

**Published:** 2019

**Authors:** George C. Rhoderick, Christina E. Cecelski, Walter R. Miller, David R. Worton, Sergi Moreno, Paul J. Brewer, Joële Viallon, Faraz Idrees, Philippe Moussay, Yong Doo Kim, Dalho Kim, Sangil Lee, Annarita Baldan, Jianrong Li

**Affiliations:** * National Institute of Standards and Technology (NIST), Gaithersburg, Maryland, US; † National Physical Laboratory (NPL), Teddington, Middlesex, UK; ‡ Bureau International des Poids et Measures (BIPM), Sévres Cedex, FR; § Korea Research Institute of Standards and Science (KRISS), Daejeon, KR; ‖ Van Swinden Laboratory (VSL), Delft, NL

**Keywords:** Volatile organic compounds, VOCs, Gas standards, Cylinder treatments, Stability

## Abstract

Measurements of volatile organic compounds (VOCs) have been ongoing for decades to track growth rates and assist in curbing emissions of these compounds into the atmosphere. To accurately establish mole fraction trends and assess the role of these gas-phase compounds in atmospheric chemistry it is essential to have good calibration standards. A necessity and precursor to accurate VOC gas standards are the gas cylinders and the internal wall treatments that aid in maintaining the stability of the mixtures over long periods of time, measured in years. This paper will discuss the stability of VOC gas mixtures in different types of gas cylinders and internal wall treatments. Stability data will be given for 85 VOCs studied in gas mixtures by National Metrology Institutes and other agency laboratories. This evaluation of cylinder treatment materials is the outcome of an activity of the VOC Expert Group within the framework of the World Meteorological Organization (WMO) Global Atmospheric Watch (GAW) program.

## Introduction

Maintaining consistent, long-term data measurement sets on volatile organic compounds (VOCs) requires sources of accurate and stable gas mixtures containing these compounds. When the World Meteorological Organization Global Atmosphere Watch (WMO-GAW) program performed comparison experiments in the 1990s and early 2000s (Schultz et al., 2015), they observed a lack of data compatibility among measurement laboratories and linked it to the inconsistency of calibration standards. The WMO approached several National Metrology Institutes (NMIs) and a working group was established. Each NMI was tasked with developing standards for a specific group of VOCs, for which they were eventually established as a Central Calibration Laboratory (CCL) and tasked with providing standards to the World Calibration Centre. It is well known among these laboratories developing and preparing VOC gas standards that a plain aluminum gas cylinder, with no special treatment to the internal cylinder walls, will not yield long-term, stable VOC gas mixtures.^1^ Several NMIs and other government agency laboratories have been preparing gaseous VOC standards in many types of cylinders, with many different types of internal cylinder wall treatments applied by specialty gas companies. The goal is to find a gas containment package that results in long-term stability, upwards of 10 years is preferred, of these VOCs in a gas mixture. Unfortunately, these internal cylinder treatments are proprietary and therefore the authors cannot comment on the actual process used in applying those treatments. We speculate that some of these treatments are chemical and others are electro-plating processes. This is unfortunate to the user as knowing those processes may help in determining why certain VOCs are stable or unstable in these cylinders. Here, we discuss some of the stability data of VOCs in gas mixtures that have been collected over decades of standards development by these NMIs.

## Analytical methods

### Analytical instrumentation

Several different types of analytical instruments were used to make measurements during the stability studies for VOCs contained in treated cylinders. Table 1 lists the different compound groups studied along with the instrumentation used to make those measurements. Detailed information on methods are too numerous to discuss in this paper. However, more detailed information can be found in the references given in Table 1 and in the discussion sections for each compound. The methods and instrumentation given in those references are identical to those used to obtain the measurement data for these stability studies.

### Regulators and sample lines

Typically, stainless steel diaphragm single or dual-stage regulators are used when sampling from the gas cylinder mixtures. For some gas mixtures considered more reactive, such as dimethyl sulfide (DMS), stainless steel diaphragm regulators (Swagelok, UK) were passivated by the Sulfinert® process (Restek Corporation, USA) to minimize the adsorption losses of trace gas compounds on the metal surfaces. Single-stage or two-stage regulators were used for the DMS analyses, depending on the amount-of-substance fractions of the target compounds and analytical conditions. Sulfinert®-treated stainless steel traps and tubing were used in the cryogenic preconcentration unit and sampling paths.

Regulators are purged with the gas mixture being sampled by closing off the exit valve on the regulator, pressurizing the regulator, and expelling the gas, several times. In most cases, regulators are removed from the cylinder after obtaining the desired number of measurements, mainly due to safety storage regulations at the NMIs. Cylinders mixtures contained in 30 L or larger volumes are typically stored in a vertical position. Smaller volume gas cylinder mixtures are stored in a horizontal position. Storage temperatures are typically around 20°C (±2°C).

### Gas cylinders and treatments

The cylinder metal type, internal treatment name and the abbreviated combination name that will be used throughout this discussion are given in Table 2. As eluded to in the Introduction, these treatment processes are proprietary and therefore we cannot describe in detail what or how they are applied to the internal cylinder surfaces. However, we do know that with the nickel-plated carbon steel cylinders the nickel is electro-plated on the walls.

The gas mixtures that will be discussed are prepared gravimetrically from pure starting materials that are analyzed and quantified for impurities. Final pressures in the gas mixtures are generally >10 MPa (1500 psi).

Several methods are used to treat measurement data. Some mixtures contain an “internal standard”, a compound known to be stable in gas mixtures such as propane, *n*-hexane or benzene. The instrument response of the VOC is divided by that of the internal standard to determine a “ratio”. The ratios are then tracked over time. Another approach to determine stability is to periodically prepare new gravimetric mixtures, the period being determined by the length of the stability testing. The new standard is then compared to the mixture being studied and the mole fractions of the VOCs determined from the fresh standards. In some cases, such as formaldehyde, primary cylinder gas standards are monitored using dynamic systems such as permeation tubes.

### Uncertainties

Typically, multiple replicate measurements are made on a gas sample when it is analyzed. For those examples given in this paper where the gas mixture contained an internal standard (*IS*), the average (*avg*) and standard deviation (*sd*) of the multiple instrument responses were calculated for each VOC and the *IS*. The *avg* response of a VOC was then divided by that of the *IS* to determine a VOC ratio (*VOC*_*r*_). The *sd* was divided by the *avg* response for both the VOC and *IS* and converted to a percent (%*sd*) unit. A combined standard uncertainty (*u*_*c*_) was then calculated using the following equation:

(1) $$u_{c}=\sqrt{{\left(VOC_{\%sd}\right)}^{2}+{\left(IS_{\%sd}\right)}^{2}}$$

The calculated analytical or measurement *u*_*c*_ was then multiplied by the *VOC*_*r*_ to determine the uncertainty, *u*_*VOCr*_

(2) $$u_{\text{VOC}r}=u_{c}\times VOC_{r}$$

which represents the error bars as seen in the figures discussed in the Results and Discussion section.

In those cases where mole fractions are discussed, rather than ratios, the mixture under stability testing is compared to an existing or new primary standard (PSM), usually gravimetrically prepared. The mole fraction of a VOC (*VOC*_*mf*_) in the test mixture is determined by dividing the average response of the VOC by that of the PSM and multiplying by the mole fraction of the PSM. Typically, the *u*_*c*_ of the VOC is calculated from the *sd* of the average of multiple responses of the VOC in the test mixture and the PSM, and the uncertainty of the mole fraction in that PSM (*uPSM*_*mf*_) expressed as % relative:

(3) $$u_{c}VOC_{mf}=\sqrt{{\left(VOC_{\%sd}\right)}^{2}+{\left(PSMS_{\%sd}\right)}^{2}+{\left(uPSM_{mf}\right)}^{2}}$$

The resulting *u*_*c*_*VOC*_*mf*_ represent the error bars illustrated in the figures discussed later on in the paper. More descriptive information on uncertainty calculations can be found in the references given in each VOC discussion section.

## Results and discussion

Over the decades of VOC gas mixture stability research, many types of cylinders and internal wall treatments have been tested by NMIs. Most of the VOCs discussed in this paper were prepared in a matrix of dry nitrogen to simplify initial research on the feasibility of developing stable VOC mixtures in cylinders. NMIs are moving towards the development of VOC standards in an air matrix but have very limited stability data currently. A few of those instances will be discussed in this paper.

Many of these cylinder types and treatments have demonstrated stability for certain compounds but not for others. The one package studied to date that demonstrates the best stability for most VOCs has been the Al-Experis cylinders from Air Products in Belgium. However, not even this cylinder/treatment is successful for all VOCs. Unfortunately, the specialty gas companies do not divulge their treatment processes to the users as they are considered proprietary. For NMIs that deliver standards and measurement services, the passivation of cylinders is the only part of the procedure that is obtained from outside the NMI as part of ISO 17034 (ISO/CASCO, 2016). NMIs are thus dependent on the consistency and cylinder-to-cylinder repeatability of the passivation treatment to deliver products within the required uncertainties. Investments in systems appropriate to conduct research and apply passivation to cylinders is costly and usually NMIs are not funded for such research. Understanding the processes applied to the cylinders, however, would assist gas chemists in determining why those treatments are successful for stability of some VOCs and not others. Having this information would then help in developing newer treatments for more specific groups of compounds.

### Non-methane hydrocarbons (NMHCs)

**Figure 1:**
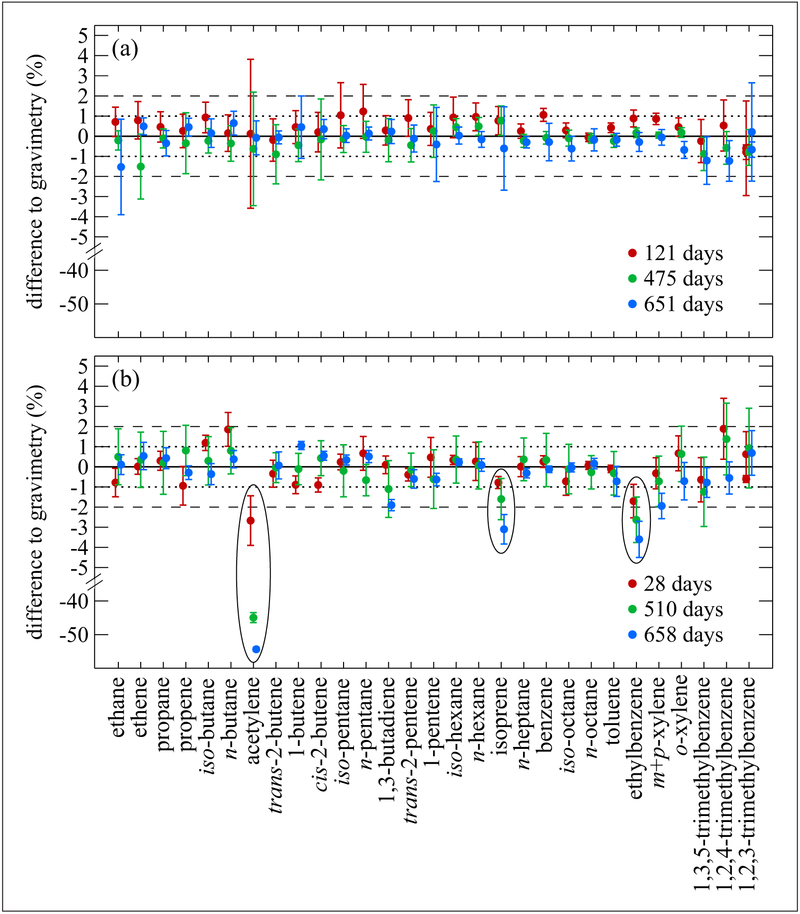
Stability of 2 NPL 30-component ozone precursor PRMs. Prepared at nominal 4 nmol mol^−1^ in Al-Experis **(a)** and Al-SS-BOC **(b)** cylinders. Measurements were made over a period of 658 days from the date of preparation. Error bars represent the expanded uncertainty, *k* = 2 (approximate 95% confidence interval), which includes contributions from the analysis and gravimetric preparation. DOI: https://doi.org/10.1525/elementa.366.f1

Several NMIs have developed gas standards containing alkanes, alkenes and aromatic hydrocarbons, and have studied those mixtures for long-term stability. The National Physical Laboratory (NPL) in the UK developed primary reference materials (PRMs) containing 30 hydrocarbons at nominal nmol mol^−1^ (ppb) mole fractions in a balance of nitrogen (Grenfell et al., 2007). These 30 hydrocarbons are recognized to be the key ozone precursor compounds as defined in European legislation 2008/50/EC (European Union, 2008). Since 2010, NPL has been the WMO CCL for 10 of these 30 non-methane hydrocarbons: ethane, propane, acetylene, *n*-butane, *iso*-butane, *n*-pentane, *iso*-pentane, isoprene, benzene and toluene. These PRMs were prepared in 10 L aluminum cylinders internally passivated with either Spectraseal (BOC) or Experis (Air Products) treatments. The PRMs were analyzed several times over a period of 2 years. For each data point a new PRM was prepared, and its response was used to assign an analytical mole fraction to the original PRM, which was compared to the gravimetric value providing a value representing the difference. Figure 1 shows this data for 2 PRMs containing the 30-component ozone precursor mixture, one prepared in an Al-Experis cylinder (Figure 1a) and another prepared in an Al-SS-BOC cylinder (Figure 1b). Overall there are no significant changes in composition for any of the 30 components in the Al-Experis cylinder. All compounds agree with the analytical and gravimetric values within 2%, most within 1%, well below the experimental uncertainties. However, there are stability issues for some of the unsaturated compounds in the Al-SS-BOC cylinder. This is most notable for acetylene, which has declined by 3% after 1 month, and by 50% after 1 year. After more than 1.5 years, isoprene and ethylbenzene show observable losses of 3% and 4%, respectively, and 1,3-butadiene and *m*+*p*-xylene show losses of 2%.

**Figure 2:**
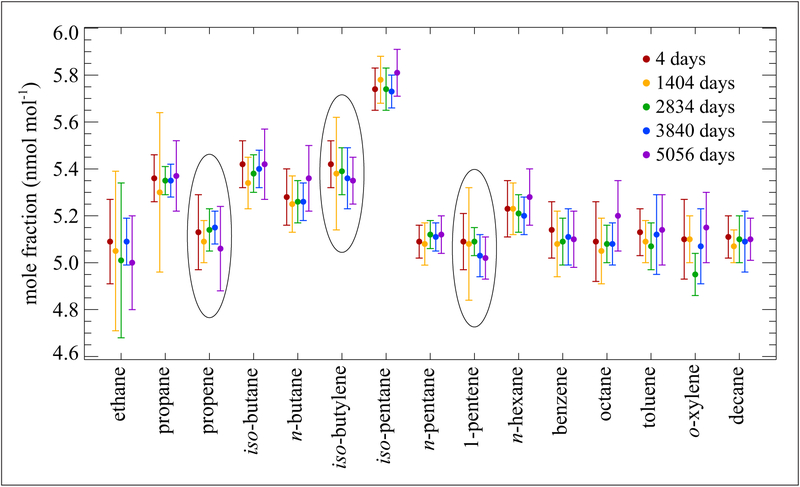
Stability of a NIST hydrocarbon SRM mixture at nominal 5 nmol mol^−1^. Contained in an Al-Acu-IV cylinder. Error bars represent the approximate 95% confidence interval. Measurements were made over a period of 5056 days from the date of preparation. DOI: https://doi.org/10.1525/elementa.366.f2

The National Institute of Standards and Technology (NIST) developed a Standard Reference Material (SRM 1800) containing 15 hydrocarbons at 5 nmol mol^−1^ (ppb) in a balance of nitrogen. The SRM mixtures were prepared in 30 L Al-Acu-IV cylinders. A few of those original SRMs have been measured at time intervals to assess their long-term stability. Figure 2 shows measurement data for one of those cylinders covering 14 years. For each data point, a minimum of one new standard was prepared to assess the stability of the original SRM sample. Overall, the data demonstrate good stability for these hydrocarbons over the 14-year period. Alkenes are circled as there may be slight losses over time, but the values are still within the 95% uncertainty limits.

**Figure 3:**
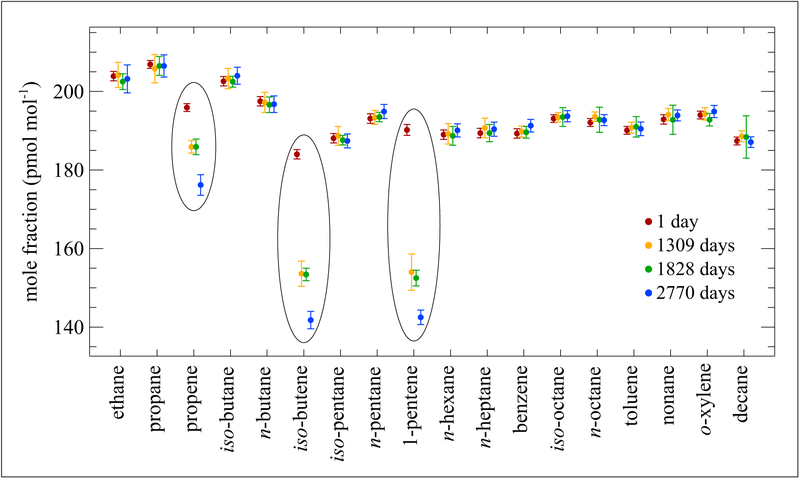
Stability of a NIST hydrocarbon mixture at nominal 200 pmol mol^−1^. Contained in an Al-Acu-IV cylinder. Error bars represent the approximate 95% confidence interval. Measurements were made over a period of 2770 days from the date of preparation. DOI: https://doi.org/10.1525/elementa.366.f3

Similar gravimetric standards containing an additional 3 hydrocarbons were developed at NIST covering a mole fraction range from (50 to 250) pmol mol^−1^ (ppt). These standards were also prepared in 30 L Al-Acu-IV cylinders. One of those standards at nominal 200 pmol mol^−1^ has been studied extensively for stability over a 7-year period as displayed in Figure 3. At the pmol mol^−1^ level the double-bonded alkenes, which are circled, show significant losses of ~20 pmol mol^−1^ to 50 pmol mol^−1^ (10% to 25% relative). This standard was used in an international comparison among these same NMIs and laboratories (Rhoderick et al., 2014). Agreement among all labs was within ±5% and the degradation of the alkenes was tracked with time as taken from all data points reported.

### Monoterpenes

**Figure 4:**
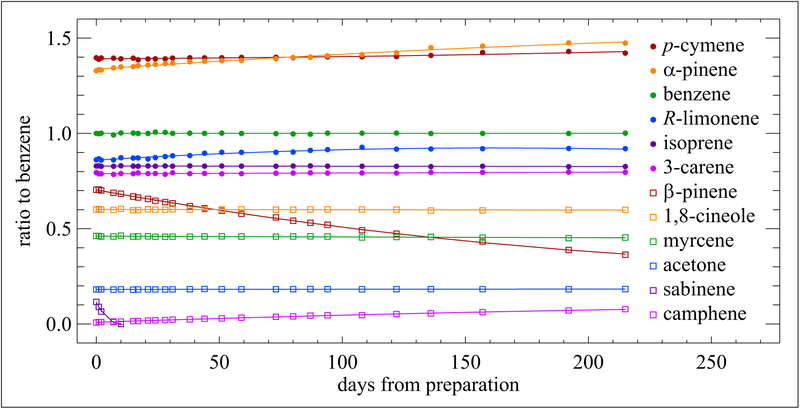
Stability of a monoterpene mixture at (3 to 10) nmol mol^−1^. Contained in an Al-Acu-IV cylinder. Measurements were made over a period of 215 days from the date of preparation. Error bars are not included here as they are too small to be visible. DOI: https://doi.org/10.1525/elementa.366.f4

Several NMIs have developed monoterpene standards and tracked their stability. Since 2013, NIST has served as the WMO CCL for monoterpenes and is responsible for developing, maintaining and disseminating the traceability for these components to WMO-GAW monitoring stations across the globe. The first cylinder/treatment combination NIST experimented with was an Al-Acu-IV cylinder. A mixture containing 9 monoterpenes, isoprene, the oxygenate acetone and benzene in a balance gas of nitrogen was prepared in a 49 L cylinder at nominal (3 to 10) nmol mol^−1^. Benzene, known to be very stable in this cylinder/treatment combination (Rhoderick, 2005), was used as an internal standard. The mixture was pre-concentrated using a Nutech pre-concentration unit coupled to a gas chromatograph (GC) equipped with a flame ionization detector (Rhoderick, 2010). For each analysis, the GC peak area of each compound was divided by the peak area of benzene; these response ratios were used to track stability. The ratio data are plotted along with linear regression or second order polynomial trend lines in Figure 4. Subtracting the 215-day ratio from the initial ratio for benzene reveals a difference of only 0.14%, which is statistically insignificant, thus illustrating stability and confirming that the cylinder itself was viable. Covering the 215 days, changes in the ratios for isoprene (−0.32%), 3-carene (−0.05%), 1,8-cineole (−0.14%) and acetone (1.1%) indicate that they were also reasonably stable. Sabinene completely disappeared in just 9 days. *β*-Pinene (−48%) and myrcene (−1.9%) degraded, while *α*-pinene (10.9%) and *p*-cymene (1.74%) increased over time. *R*-limonene (6.8%) showed a steady initial growth rate, but that rate appeared to slow down at ~100 days. Camphene, which consistently increased in the mixture over time (~589%), was not intentionally added to this mixture, but was initially present in very small amounts as an impurity from some of the other monoterpenes. The possibility exists that initial losses occurred when the monoterpenes are introduced into the cylinder at preparation. These would not have contributed to the percent losses discussed here, but would be additional indeterminable losses.

It appears that a chemical transformation was taking place among the monoterpenes in the mixture and not necessarily an instability issue due to interactions of the compounds with the cylinder wall. Previous research on the isomerization of gas-phase *β*-pinene over acid-activated bentonite revealed that there are reaction products of *α*-pinene, *R*-limonene and camphene (Foletto et al., 2002). A few studies indicate isomerization of *α*-pinene to mainly camphene and some limonene (Allahverdiev et al., 1998; Findik and Gündüz, 1997). Even though these studies were done under high temperature, it may be that chemical transformations of the *β*-pinene and *α*-pinene in the presence of oxides could take place at ambient temperature. Most likely aluminum oxide (Al_2_O_3_) is present in the gas cylinder, which could be the catalyst for the transformation of *β*-pinene into *α*-pinene, *R*-limonene and camphene, as well as other monoterpenes. Similarly, the *α*-pinene could react with any Al_2_O_3_ present in the cylinder to form other monoterpenes and eventually result in a decrease of *α*-pinene. It is most probable that there are molecules of Al_2_O_3_ attached to the cylinder walls. Therefore, to develop a stable monoterpene mixture, a cylinder treatment would be needed to create a barrier between the cylinder walls and the gas mixture.

**Figure 5:**
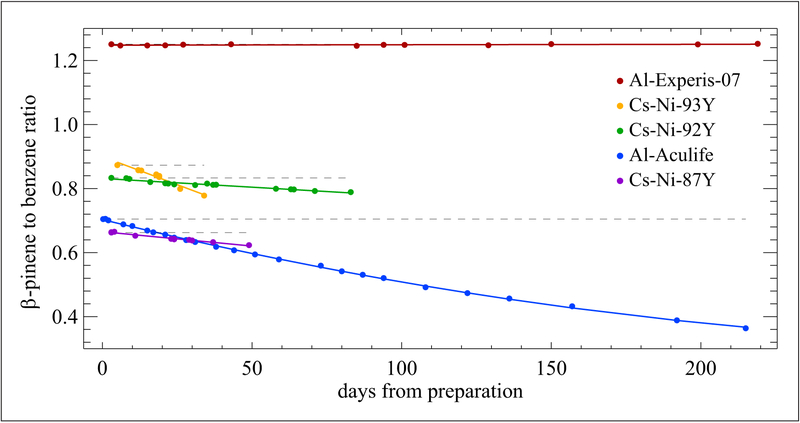
Stability of *β*-pinene at nominal 5 nmol mol^−1^. Contained in different cylinder/treatment packages as shown in the legend. Dotted lines represent a linear extrapolation from the first ratio data point. Error bars are not included as they are too small to be visible. DOI: https://doi.org/10.1525/elementa.366.f5

Two other cylinder/treatment packages were studied to determine if stable gas mixtures of monoterpenes could be developed. One Al-Experis and three CS-Ni cylinders were tested along with one Al-Acu-IV cylinder. Mixtures were prepared containing different combinations of monoterpenes at nominal 5 nmol mol^−1^ (Rhoderick and Lin, 2013). The CS-Ni cylinder mixtures all contained *β*-pinene but no *α*-pinene, and each mixture contained a few other monoterpenes. All 3 CS-Ni mixtures displayed losses of *β*-pinene at different rates over the periods studied, with an average total loss of 8.5%. There was an average growth of *α*-pinene, not added intentionally to the mixtures, of 21%. Also, there were losses of 3-carene and 1,8-cineole, averaging 19% and 15.6%, respectively. The Al-Experis cylinders showed very consistent stability over the 215-day period. Figure 5 summarizes the *β*-pinene stability results for each of the cylinder/treatment packages tested. As shown, the Al-Experis cylinder is the only cylinder for which *β*-pinene is stable.

**Figure 6:**
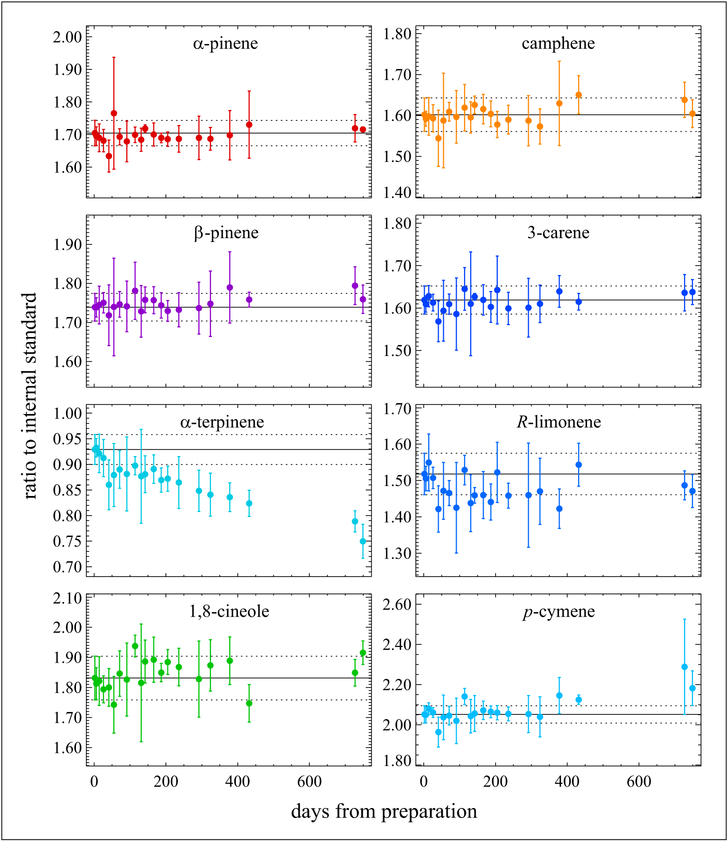
Stability of monoterpenes in air at nominal 2 nmol mol^−1^. Contained in an Al-Experis cylinder. Measurements were made over a period of about 800 days from the date of preparation. The solid black line represents the first ratio determined for a monoterpene to the internal standard (*n*-hexane), with the corresponding dotted lines marking the *k* = 2 expanded uncertainty. Error bars for each ratio represent *k* = 2 expanded uncertainties. DOI: https://doi.org/10.1525/elementa.366.f6

Additional standards have been developed in Al-Experis cylinders containing different combinations of monoterpenes in nitrogen at nominal 2 nmol mol^−1^. The analytical data from those studies show good stability beyond 3 years for many monoterpenes, including *β*-pinene. One monoterpene standard in an air matrix at 2 nmol mol^−1^ has been tracked for stability for nearly 800 days as shown in Figure 6. Six of the 8 monoterpenes demonstrate good stability with a threshold change of <0.5% yr^−1^, whereas *α*-terpinene exhibits significant losses (−19%) over that time; *p*-cymene appears to be increasing in mole fraction but is still considered within the 0.5% yr^−1^ threshold.

**Figure 7:**
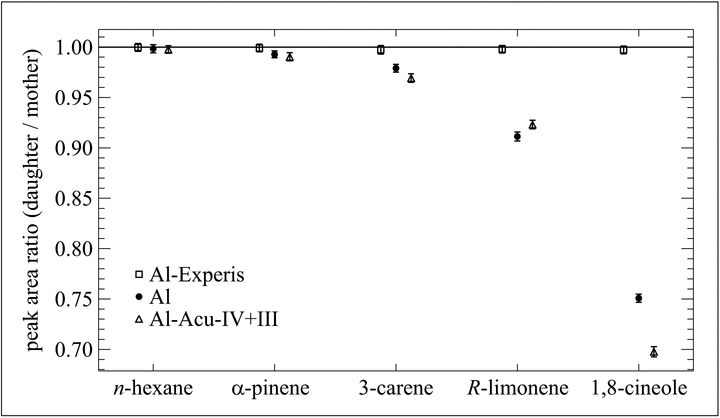
Adsorption losses of monoterpenes at 2.5 nmol mol^−1^. Contained in 3 different aluminum cylinders. Losses are determined from mother-to-daughter testing over a period of days. Error bars represent the approximate 95% confidence interval. DOI: https://doi.org/10.1525/elementa.366.f7

The Korea Research Institute of Standards and Science (KRISS) has developed and studied the stability of monoterpene standards at nominal 2.5 nmol mol^−1^. They studied mixtures in 3 different types of cylinders with different treatments: 10 L Al (no treatment), 10 L Al-Experis, and 6 L Al-Acu-IV+III (Kang et al., 2016). Each cylinder was prepared with a mixture containing *α*-pinene, 3-carene, *R*-limonene, 1,8-cineole, and *n*-hexane (as an internal standard) in a balance of nitrogen. Immediate physical adsorption loss on the internal cylinder surface was evaluated using cylinder-to-cylinder division (mother-to-daughter) (Lee et al., 2017) for each type of cylinder. Results from these studies, depicted in Figure 7, show the same type of results as the NIST studies. There was no adsorption loss of the 5 components in the Al-Experis cylinder. Those data are based on peak area ratios of mother-to-daughter cylinders (as described in Lee et al. (2017)) and were not different from a ratio 1.000 within the analytical uncertainties (±0.2%). In contrast, for both the Al-Acu-IV+III and Al cylinders, there were significant adsorption losses for all compounds except *n*-hexane as the peak area ratios were different from 1.000. The adsorption losses of the oxygenated 1,8-cineole were notably larger than those of the other monoterpenes.

Stability of these mixtures was then monitored over a period of 45 days, by tracking peak area ratios of each monoterpene to *n*-hexane. During this study, the peak area ratios for all monoterpenes agreed within the analytical uncertainty of ~1% for the Al-Experis cylinder mixture. In the Al cylinder, however, peak area ratios of *α*-pinene, 3-carene, *R*-Limonene and 1,8-cineole decreased by 10.4%, 1.7%, 15.5% and 22.8%, respectively. In the Al-Acu-IV+III cylinder, *α*-pinene showed no distinctive trend, whereas 3-carene, *R*-limonene, and 1,8-cineole showed strong decreases of 1.9%, 6.5% and 15.5%, respectively.

### Formaldehyde

While several papers can be found in the literature on dynamic generation of formaldehyde standards (Brewer et al., 2013; Chu et al., 1997; Ho, 1985), preparation of static standards at the low levels found in the atmosphere (around 0.1 nmol mol^−1^) appears to be very challenging. Attempts to fill cylinders at the μmol mol^−1^ (ppm) levels started within NMIs after 2000, with first measurements reported by Brewer et al. (2013) showing formaldehyde losses of 0.2% over 9 months using a 10 L Al-SS-BOC cylinder.

**Figure 8:**
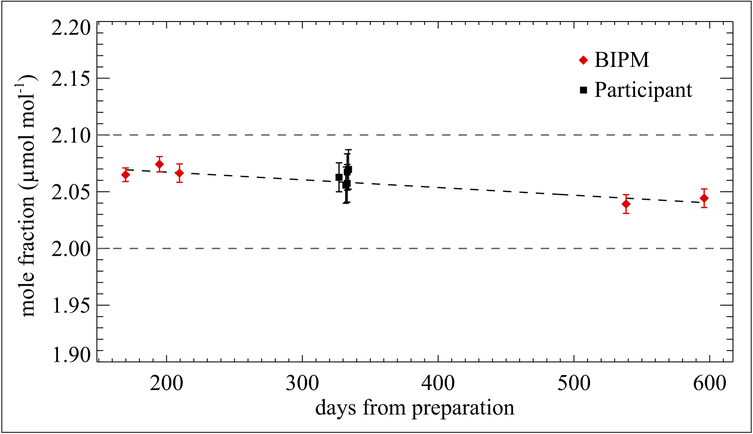
Mole fraction of formaldehyde in nitrogen. Contained in one of the 30 L Al-Acu-VIII cylinders used as a transfer standard in the key comparison CCQM-K90. Measurements were made over a period of 600 days from the date of preparation. Red diamonds indicate measurements performed at the BIPM and black squares indicate measurements performed by the NMI participants in this key comparison. Error bars represent the approximate 95% confidence interval. DOI: https://doi.org/10.1525/elementa.366.f8

**Figure 9:**
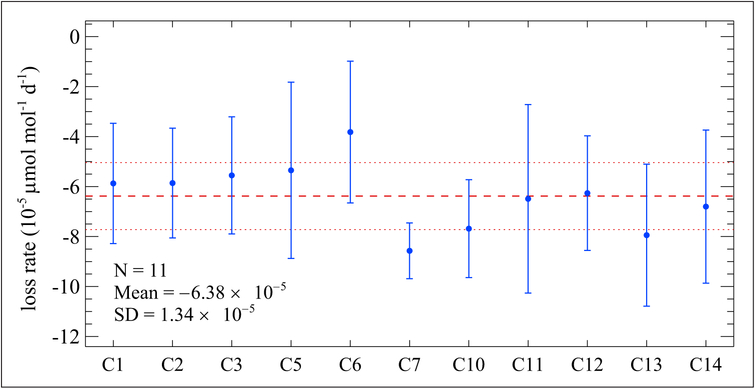
Average loss per day of formaldehyde in nitrogen. At a nominal mole fraction of 2 μmol mol^−1^ in a series of 30 L Al-Acu-VIII cylinders; C1, C2, etc. denotes individual cylinders. Measurements were made over a period of 600 days from the date of preparation. Error bars represent *k* = 2 expanded uncertainties for each data point. The mean and standard deviation of all 11 cylinders are represented by the dashed and dotted red lines, respectively. DOI: https://doi.org/10.1525/elementa.366.f9

The Bureau International des Poids et Mesures (BIPM; Sèvres, France) and KRISS have also studied the stability of formaldehyde in nitrogen gas mixtures. In preparation of the international key comparison CCQM-K90, Formaldehyde in nitrogen at nominal 2 μmol mol^−1^ (Viallon et al., 2017), the BIPM tested 3 cylinders provided by 3 different facilities of Air Liquide. Of the 3, only 1 facility included a treatment process with their cylinder, which was Al-Acu-VIII; this was the only cylinder provided by Air Liquide that showed an acceptable stability. (No commercial treatment name was given for the other 2 cylinders that failed; they were abandoned as they were not successful). As a result, 14 Al-Acu-VIII cylinders containing formaldehyde at a nominal mole fraction of 2 μmol mol^−1^ were obtained and used as transfer standards during the key comparison CCQM-K90. For the aim of the comparison, the formaldehyde mole fractions were evaluated regularly at the BIPM before and after the measurement by each participant. Measurements were performed with Fourier transform infrared (FTIR) spectroscopy and calibrated based on continuous weighing of 2 different dynamic generation sources: a permeation tube containing paraformaldehyde and a diffusion cell containing trioxane. Figure 8 shows that one of the cylinders experienced a linear loss rate, allowing an easy estimation of the formaldehyde mole fraction as measured by the BIPM at any time during the on-site analysis. This was essential for the calculation of degrees of equivalence, the agreement between an NMI value and the Key Comparison Reference Value (KCRV) determined for all NMI data points in this comparison. The formaldehyde loss was estimated for 11 cylinders in the comparison and found to be, on average, −1.9% over 600 days, remarkably similar for all cylinders as shown in Figure 9. Al-Acu-VIII cylinders thus appear to be suitable for use with formaldehyde in nitrogen mixtures, with a relative loss rate of <1% yr^−1^, moreover easily predictable using a linear model.

**Figure 10:**
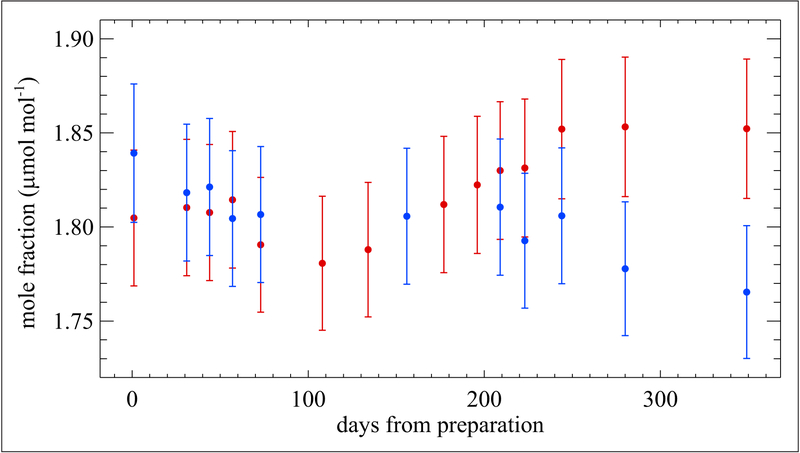
Long-term stability of 2 μmol mol^−1^ formaldehyde in nitrogen standards. Contained in 2 Al-Acu-IV+III cylinders, as depicted by the different colors. Measurements were made over a period of 365 days from the date of preparation. Error bars represent the approximate 95% confidence interval. DOI: https://doi.org/10.1525/elementa.366.f10

KRISS has developed and studied stability for standards of formaldehyde in nitrogen at 2 μmol mol^−1^ levels in 10 L Al (untreated), 10 L Al-Experis, and 6 L Al-Acu-IV+III cylinders. Two standards were prepared for each type of cylinder/treatment combination with similar mole fraction levels, monitoring their long-term stabilities for 365 days. The data for the Al-Experis and Al cylinders showed linear decreasing trends over the 365 days of −2.5% and −0.7%, respectively. The formaldehyde gas mixtures in the Al-Acu-IV+III cylinders showed stability, threshold of <1%, for 1 year as illustrated in Figure 10.

### Oxygenated VOCs (OVOCs)

OVOCs are analytically challenging components to measure due to their tendency to adsorb to surfaces (Rhoderick, 1998). This can lead to memory effects, in which the analysis of one mixture is affected by residuals leftover from a previously analyzed mixture. As a result, long-term conditioning of flow paths is necessary to ensure a stable response. The ideal approach for monitoring stability of OVOCs is through the preparation of new mixtures at each time step, or use of in-situ gas mixture generation (dynamic methods such as diffusion or permeation) to avoid losses due to adsorption on contact surfaces. However, this is more labor intensive and expensive compared to the use of an internal reference. Great care is taken to minimize differences by using the same GC system, adopting standard operating procedures for flow rates and purging times, and by ensuring that the response of the OVOCs of interest are stable before measurements are taken. In this way, the influence of analytical artifacts is assumed to be reduced—or at least systematically constant in time—and therefore facilitating the evaluation of cylinder stability issues.

**Figure 11:**
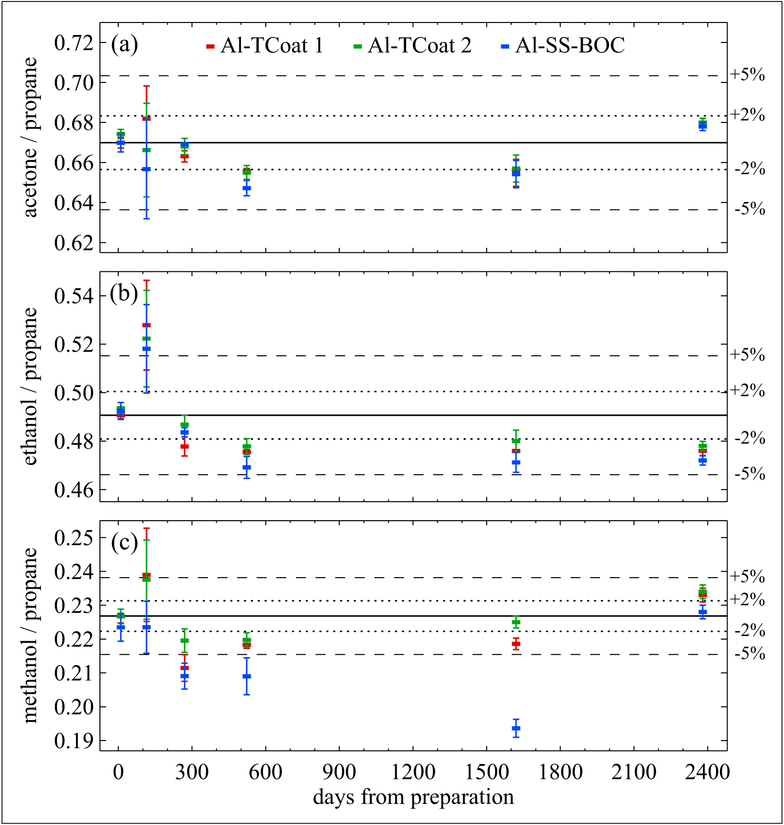
Stability of acetone (a), ethanol (b) and methanol (c) at nominal 5 μmol mol^−1^ relative to propane. Prepared in 10 L Al-TCoat and 5 L Al-SS-BOC cylinders. Measurements were made over a period of 2400 days from the date of preparation. Error bars represent combined standard uncertainties, i.e., standard deviations of multiple replicate measurements. DOI: https://doi.org/10.1525/elementa.366.f11

Several NMIs have developed standards for various OVOCs. Van Swinden Laboratory (VSL) has developed OVOCs gas standards containing methanol, ethanol, acetone and propane (the latter was used as an internal standard) at (1 to 10) μmol mol^−1^ levels in nitrogen in Al-SS-BOC (5 L) and Al-TCoat (10 L) cylinders. Before this, VSL investigated the feasibility of preparation in Al-Acu-IV and Al-Experis cylinders. A 1-year study showed that Al-Experis cylinders were suitable for acetone but yielded losses for methanol (−10%) and ethanol (−2%) at 1 μmol mol^−1^. For Al-Acu-IV cylinders, an immediate loss after preparation was found for all components, respectively 5% for acetone and 15% for methanol and ethanol. This loss increased over time for methanol, while not changing for acetone or for ethanol. VSL then prepared 2 gas mixtures of acetone, methanol and ethanol in 5 L Al-SS-BOC cylinders and 3 gas mixtures in 10 L Al-TCoat cylinders, all at at 5 μmol mol^−1^. Stability of these mixtures was monitored for more than 6 years, using propane as an internal standard (propane has demonstrated stability in these types of cylinders). Figure 11 reports the stability data for one of the Al-SS-BOC cylinders and for 2 of the Al-TCoat cylinders. Like in cases described above by other institutes, the ratios of the GC peak area of each OVOC to the propane peak area have been used for measuring the degree of stability. The error bars indicate the repeatability of measuring the OVOCs with the GC system. The scattering of the points in the same figure, particularly for methanol, indicates the difficulty of obtaining properly reproducible results with the GC system. As shown, the degree of stability for these OVOCs at this mole fraction is within their analytical uncertainty: 2% relative for acetone, 3% for ethanol and 5% for methanol. The measurement data for methanol in Al-SS-BOC cylinders are less consistent, and this is probably due to the purging time of the gas sample before analysis, which was improved in the last analysis.

**Figure 12:**
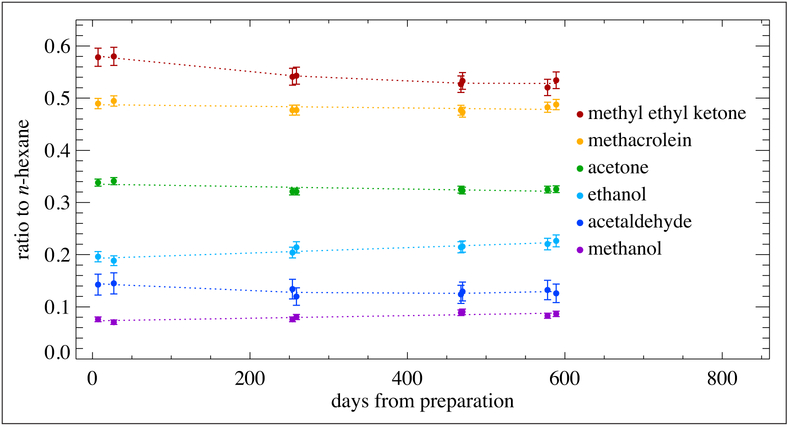
Stability of OVOCs at nominal 100 nmol mol^−1^. Contained in a 3.6 L SS-Silco cylinder. Measurements were made over a period of 600 days from the date of preparation. Error bars represent the approximate 95% confidence interval. DOI: https://doi.org/10.1525/elementa.366.f12

After the development of OVOC gas standards at (1 to 10) μmol mol^−1^ levels, VSL targeted a new set of OVOC gas standards at 100 nmol mol^−1^, which contained methanol, ethanol, acetone, methyl ethyl ketone, methyl vinyl ketone, methacrolein and acetaldehyde (with propane and *n*-hexane as internal standards) in nitrogen. These standards were prepared in SS-Silco cylinders (3.6 L) and in 4 different aluminum cylinders (10 L) with various proprietary treatments. The SS-Silco cylinders demonstrated the best ability to minimize the interaction with OVOCs. The stability behavior of the OVOC gas mixtures in the 3.6 L SS-Silco cylinders is expressed as a ratio of the OVOC GC responses to *n*-hexane and is represented in Figure 12. Acetone and methacrolein remain stable within 5% over a period of ~20 months (7300 days). Methyl ethyl ketone and acetaldehyde show a decrease at 6 months (between −5% and −10%), after which they remain stable. The ongoing studies on a new set of PRMs will show if this effect is reproducible or if it is caused by changes in analytical procedure. For ethanol and methanol, the stability results show an increase of mole fractions (~10%) which is explained by changes in the analytical procedure at 6 months and by a significant decrease of gas pressure over time due to the small cylinder volume rather than by reaction effects.

**Figure 13:**
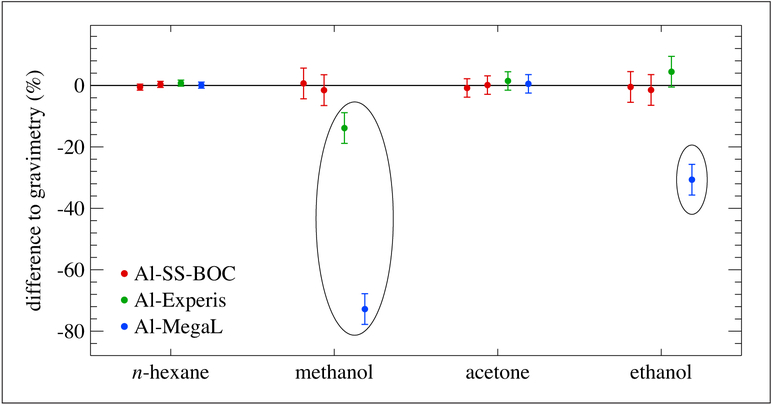
Preparation losses for alcohols in 3 cylinder treatments. Measurements were made starting 1 day from the date of preparation. Error bars for each data point represent the approximate 95% confidence interval. DOI: https://doi.org/10.1525/elementa.366.f13

NPL has focused on PRMs containing methanol, ethanol and acetone due to their importance in atmospheric chemistry and to the WMO-GAW program. NPL investigated the influence of 3 different cylinder passivation treatments (Al-SS-BOC, Al-Experis and Al-MegaL) on 10 L standards containing methanol, ethanol, acetone and *n*-hexane at nominal mole fractions of 100 nmol mol^−1^ in nitrogen. These were prepared in accordance with ISO 6142 as a single step dilution of a 5 μmol mol^−1^ parent mixture. Figure 13 shows results for all 3 cylinder types with consistent results of *n*-hexane and acetone but not for the primary alcohols. Methanol was lost substantially on preparation in Al-Experis (−15%) and in Al-MegaL (−75%). Ethanol also showed significant loss on preparation in the Al-MegaL cylinders (−30%). It is NPL practice not to use cylinders that show large initial losses due to concerns over what happens to that adsorbed material during the use of the cylinder. In a recent paper, Brewer et al. (2018) have shown that at low pressures (<25 bar), desorption of previous adsorbed gases (carbon dioxide and hydrochloric acid) can affect the mole fraction of reference materials.

**Figure 14:**
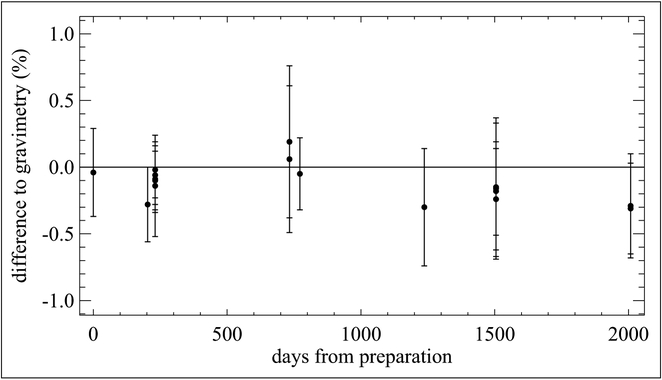
Stability of 5 μmol mol^−1^ *n*-hexane mixtures. Contained in 10 L Al-SS-BOC cylinders. Measurements were made over a period of 2000 days from the date of preparation. Error bars represent *k* = 2 expanded uncertainties. DOI: https://doi.org/10.1525/elementa.366.f14

**Figure 15:**
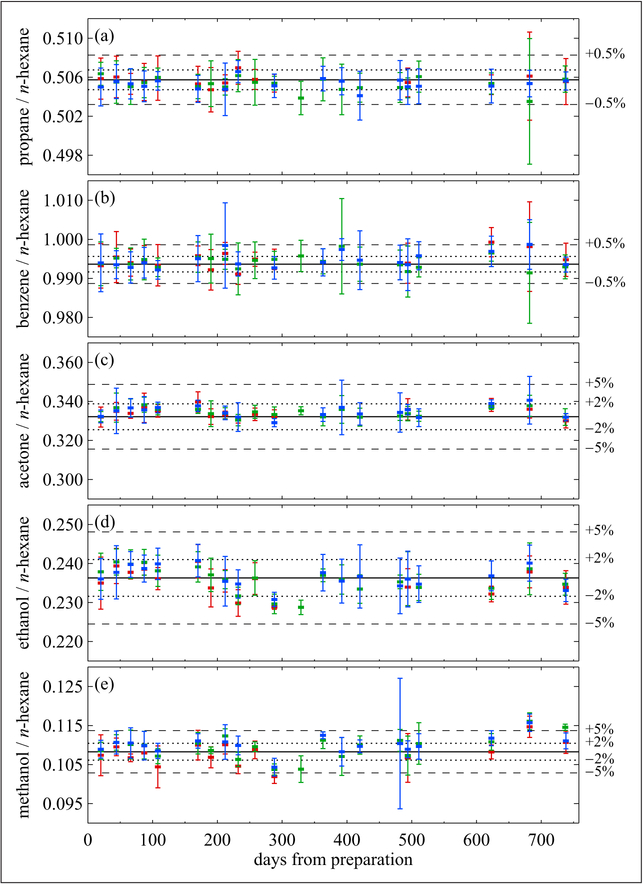
Stability of OVOCs at nominal μmol mol^−1^. Response ratios of propane **(a)**, benzene **(b)**, acetone **(c)**, ethanol **(d)** and methanol **(e)** to *n*-hexane for 3 individually prepared mixtures (as depicted by the different colors), each contained in an Al-SS-BOC cylinder. Measurements were made over a period of approximately 750 days from the date of preparation. The error bars, which represent the *k* = 2 expanded uncertainties, are related to the component in an individual mixture, and do not depict the difference between cylinders. DOI: https://doi.org/10.1525/elementa.366.f15

Following this, NPL prepared a batch of 3 standards, each individually prepared containing methanol, ethanol, acetone, *n*-hexane, propane and benzene at nominal mole fractions of 5 μmol mol^−1^ in a balance of nitrogen in 10 L Al-SS-BOC cylinders. Each mixture was analyzed approximately every month for 2 years. Hexane, shown to be stable in this cylinder type for ≥5 years (Figure 14), was used as an internal standard to track stability. All compounds in the mixtures were observed to be stable over a 2-year period (~700 days) as shown in Figure 15. The propane/*n*-hexane and benzene/*n*-hexane trends were very consistent with variations of less than 0.5% from the starting values (Figure 15a and b). The OVOC/*n*-hexane ratios showed a great degree of scatter relative to the hydrocarbons. There is very good agreement among all 3 cylinders within each time point; the observed variability between each time point is likely the result of instrumental conditioning of lines and valves, and highlights one of the many challenges of measuring these types of compounds.

### Dimethyl sulfide (DMS)

KRISS has developed DMS standards at (0.5 to 7) nmol mol^−1^ levels in Al-Experis cylinders. Prior to the development of DMS standards in the Al-Experis cylinders, mixtures containing propane, benzene, and DMS were prepared in a nitrogen balance in 3 different types of cylinders with different treatments: 10 L Al (untreated), 10 L Al-Experis, and 6 L Al-Acu-IV+III (Kim et al., 2018a). Immediate physical adsorption loss on the internal cylinder surface was evaluated using cylinder-to-cylinder division for each type of cylinder. The peak area ratios of mother-to-daughter cylinder testing were not different from a ratio of 1.00 (±1.0%), which is within the analytical uncertainties. Results from these studies show that there was no adsorption loss of DMS in the Al-Experis cylinder, which was not observed in the other types of cylinder treatments. In contrast, for both the Al-Acu-IV+III and Al cylinder mixtures there were significant adsorption losses of DMS, with ratios less than 0.96 and 0.72, respectively. Longer term stability of 7 nmol mol^−1^ DMS in Al-Experis cylinders was also monitored by tracking the peak area ratio of DMS to that of benzene (internal standard) for 180 days. Results from these stability studies showed that the peak area ratios agreed within the analytical uncertainties (typically less than about 1%). The DMS in the Al-Experis cylinder was projected to be stable for more than 4 years within an uncertainty of 3% as the predicted ratio was within 3% of the initial ratio. Stability of (0.5 to 7) nmol mol^−1^ DMS in Al-Experis cylinders was evaluated by comparing against fresh DMS standards generated from a dynamic dilution method (DDM) (Kim et al., 2016) about 10 months after the gas standards were prepared gravimetrically. Results showed that DMS standards at 2 nmol mol^−1^, 5 nmol mol^−1^, and 7 nmol mol^−1^ agreed with freshly generated DMS by DDM within gravimetric uncertainties assigned to those standards. The 0.5 nmol mol^−1^ standard deviated from the fresh DMS by about −5.4% (outside of its associated uncertainty), indicating that DMS standards at <1 nmol mol^−1^ are not stable even for 10 months (Kim et al., 2018b).

### Halocarbons

**Figure 16:**
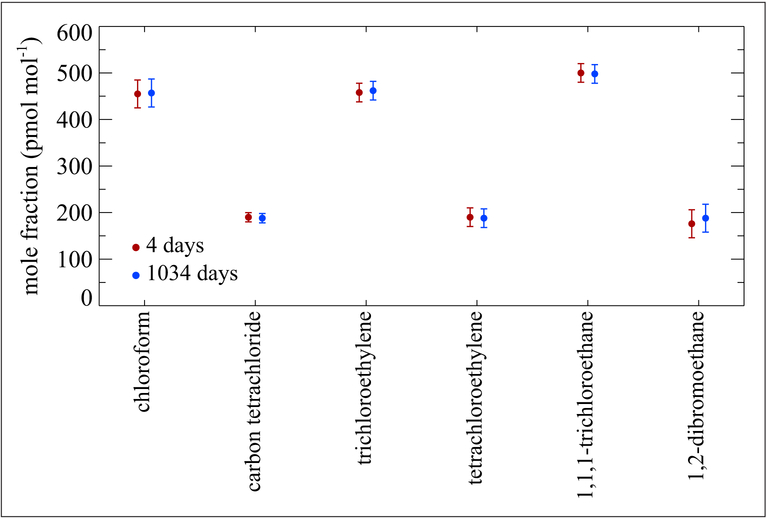
Stability of halogenated compounds at (200 to 500) pmol mol^−1^. Contained in a 3.4 L aluminum (old alloy) Aculife IV treated cylinder. Error bars represent the approximate 95% confidence interval. Measurements cover a 2.8-year time period. DOI: https://doi.org/10.1525/elementa.366.f16

**Figure 17:**
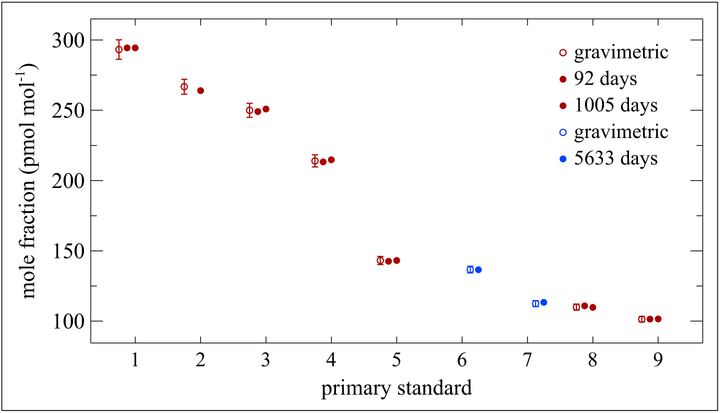
Stability of carbon tetrachloride at (100 to 300) pmol mol^−1^. Contained in 3.4 L aluminum (old alloy) Aculife IV treated cylinders. Primary standards marked in red were prepared in January 1988; those in blue were prepared in June 1989. Open circles represent gravimetric values, and closed circles represent measurements taken on the indicated number of days from the date of preparation. Error bars represent the approximate 95% confidence interval. DOI: https://doi.org/10.1525/elementa.366.f17

Several NMIs and other major atmospheric research laboratories have been developing halocarbon standards at ambient mole fraction levels (pmol mol^−1^) for decades. Both the National Oceanic and Atmospheric Administration (NOAA; https://www.esrl.noaa.gov/gmd/) and the Advanced Global Atmospheric Gases Experiment (AGAGE; https://agage.mit.edu/) have maintained halocarbon standard scales for many years that have been used extensively for atmospheric measurements of these compounds. Two NMIs have compared their standards for comparability for 6 key halocarbons through key comparison CCQM-K83 (Rhoderick, Guenther, Duewer, Lee, Moon et al., 2014). Several different cylinder/treatment packages have been studied. NIST’s original halocarbon standards were developed in 3.4 L Aculife IV cylinders in an air matrix. An example of the stability of these halogenated compounds at a nominal mole fraction of (200 to 500) pmol mol^−1^ is shown in Figure 16. While the time frame is just under 3 years, the data illustrate good stability. Figure 17 shows stability data for 9 standards of carbon tetrachloride in air at mole fractions of (100 to 300) pmol mol^−1^ contained in 3.4 L Aculife IV cylinders. Good stability is shown over a 2-year period with 2 of the mixtures exhibiting stability over a 16-year period. Of note is that these mixtures were contained in pre-1990 aluminum alloy cylinders. Aluminum manufacturers changed the alloy in the 1990s to address a weakness in the older alloy that caused cracks in the neck of the cylinders. However, NIST has observed instability of carbon tetrachloride mixtures, at pmol mol^−1^ levels, using the newer aluminum alloy cylinders treated with Aculife IV in all sizes: 3.4 L, 6 L and 30 L (not shown).

**Figure 18:**
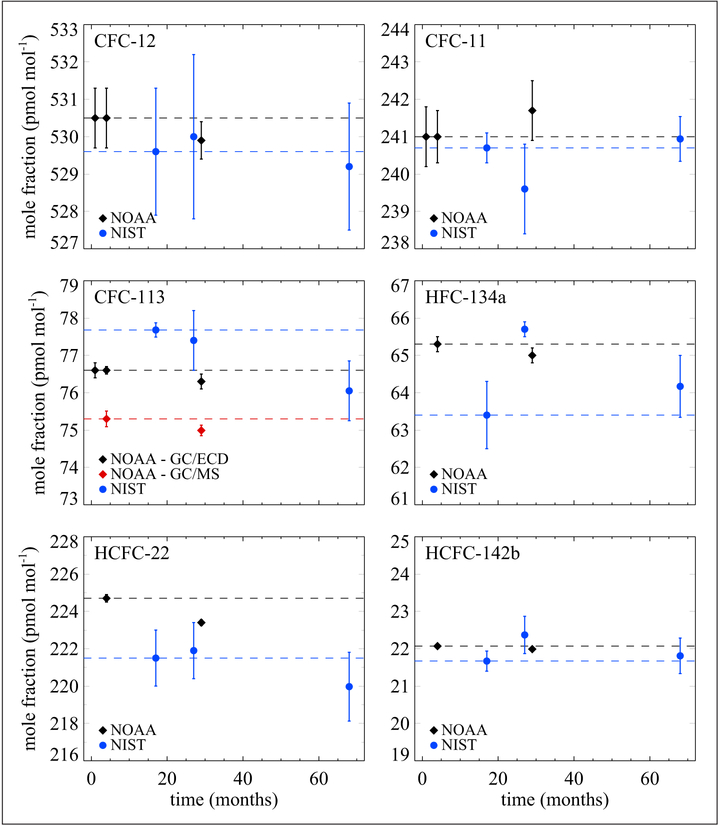
Stability of key halocarbons at near ambient levels. Contained in a 30 L Al-Acu-IV cylinder. Error bars represent combined standard uncertainties. DOI: https://doi.org/10.1525/elementa.366.f18

NIST has explored the stability of a gas mixture in a balance of air containing ambient levels of some key halocarbons: dichlorodifluoromethane (CFC-12), trichlorofluoromethane (CFC-11), 1,1,2-trichlorotrifluoroethane (CFC-113), 1,1,1,2-tetrafluoroethane (HFC-134a), difluorochloromethane (HCFC-22), and 1,1-difluoro-1-chloroethane (HCFC-142b). Over a 6-year period, NOAA and NIST have made measurements on this cylinder. Figure 18 shows the data on those measurements illustrating the stability of those halocarbons contained in a 30 L Al-Acu-IV (new alloy) cylinder.

### Other VOCs

NIST has been developing standards of key VOCs for decades and has many examples of stability in treated aluminum cylinders (Miller and Rhoderick, 1995; Rhoderick and Yen, 2006). An example of VOC stability at the nominal 5 nmol mol^−1^ level in a 30 L Al-Acu-IV cylinder is given in Table 3. Most of the compounds have shown good stability for 13 years. Carbon tetrachloride, 1,1,1-trichloroethane, trichloroethylene, 1,2-dibromoethane, ethylbenzene and *o*-xylene, in italics, start to show losses outside of the uncertainty limits somewhere between 3 and 13 years. Bromomethane and 1,3-butadiene, also in italics, likely begin to show losses shortly after preparation.

NIST has also tested 30 L Al-SpecShield cylinders for VOC mixture stability. An example of stability for select VOCs at nominal 1 nmol mol^−1^ is given in Table 4. As in the Al-Acu-IV cylinder, 1,3-butadiene, bromomethane and 1,2-dibromoethane, in italics, show losses over time. Losses are also observed for carbon tetrachloride, *cis*/*trans*-1,3-dichloropropenes and styrene, denoted by italics.

### Stability as a function of pressure

The authors did not address how the gas pressure in the cylinder affects stability, except for a mention in the section on oxygenated VOCs. It has been observed that many types of compounds, including VOCs, will show increases or decreases in mole fraction as the gas pressure decreases. There has been much interest of late in the initial adsorption of carbon dioxide (CO_2_) in air balance mixtures from the initial filling of a cylinder followed by desorption as the pressure in the cylinder is reduced, resulting in an increase in the mole fraction of CO_2_ (Leuenberger et al., 2015; Miller et al., 2015). This adsorption/desorption can be modeled for different cylinder volumes using the Langmuir isotherm (Langmuir, 1916, 1918). The NMIs have not actually dedicated significant time to controlled mole fraction determinations at specific decreasing gas pressures for VOCs as in these studies. However, it has been observed in some cases that both decreases and increases in mole fractions of VOCs have occurred once the pressure in a cylinder is reduced to a range of 1 MPa to 3 MPa. Acetone and methyl ethyl ketone are examples, as discussed in *Oxygenated VOCs (OVOCs)*, that appear to be attributed to Langmuir isotherms. Some VOCs, in particular alkanes, have been observed to remain stable with cylinder pressure exhaustion, but this can also be cylinder dependent.

## Summary and conclusions

All the stability data for the 85 VOCs discussed have been condensed into a summary found in Tables 5a, 5b, 5c and 5d. The different cylinders/treatments, the VOCs, and the stability threshold or rate losses and gains are shown in the table. All rate losses given assume linear losses, given in % yr^−1^, in order to compare all data on a common unit. However, many losses exhibit non-linear behavior, and the authors warn that these rates should only be viewed as a very general guideline as to changes that occur over not just a single year but many. Additionally, we extrapolate linearly to a year rate if the percent loss or gain is based on data covering less than a year. Some of the VOCs may have been tested in the same cylinder/treatment type using pre-1990 alloy cylinders and post-1990 alloy and show different results. Some of these data show that the alloy used may also be a factor in stability.

Stability studies of NMHCs, OVOCs and halogenated VOCs in dry air or nitrogen contained in high pressure cylinders were performed in the last decades by 5 different laboratories, on time scales ranging from 1 to more than 15 years. A total of about 85 different VOCs were looked at, with mole fractions as low as a few pmol mol^−1^ for some compounds (halocarbons, some hydrocarbons), a few nmol mol^−1^ for most of them, up to a few μmol mol^−1^ for the most unstable compounds (formaldehyde, some alcohols). Thirteen different types of cylinders with their specific internal wall treatments were tested during these studies, including 11 made from aluminum and 2 from steel.

The stability data gathered and discussed here show that a large majority of VOCs can be stable in at least one type of cylinder at an appropriate concentration. However, there is no single cylinder/treatment combination that will work for virtually any VOC. Data also show that one type of treatment appears to provide good stability for a large fraction of VOCs, and authors regret that the specialty gas companies will not divulge their treatment processes to the users as they are considered proprietary. Understanding the process applied to the cylinders would benefit research in this area, allowing specialized laboratories to prepare gas mixture standards with the appropriate level of concentration and stability to underpin more VOC measurements.

Most of the VOCs discussed here have been studied for stability in more than 1 cylinder with the same treatment. In general, the same stability or rates of losses are consistent from cylinder to cylinder with the same treatment. However, 2 cylinders with the same treatment containing the same compound mixture may not show the same stability. Examples of compounds exhibiting stability in one but not another are carbon tetrachloride and 1,1,1-trichloroethane. Manufactures warn (through verbal communications) that there could be a stability failure rate of around 25% for a batch of cylinders with the same treatment.

Since cylinder treatments are proprietary, the users cannot learn how those treatments possibly contribute to VOC stability or not. An option moving forward would be for the NMIs themselves to begin research into cylinder treatments. However, this is a very expensive proposition, demanding time and funding which most NMIs would not be able to support.

## Figures and Tables

**Table 1:** Analytical instrumentation used to study stability for different compound groups. DOI: https://doi.org/10.1525/elementa.366.t1

Compound group	Analytical instrumentation^a^	Reference
Non-methane hydrocarbons	GC-FID/preconcentration	Grenfell et al., 2007; Rhoderick, 2005
Monoterpenes	GC-FID/preconcentration	Rhoderick, 2010; Rhoderick and Lin, 2013; Kang et al., 2016; Lee et al., 2017
Formaldehyde	CRDS; FTIR	Brewer et al., 2013; Viallon et al., 2017; Panda et al., 2016
Oxygenated VOCs	GC-FID	Apel et al., 1995; Englert et al., 2018
Dimethyl sulfide	GC-FID/preconcentration	Kim et al., 2016, 2018a
Halocarbons	GC-ECD; GC-MS/preconcentration	Rhoderick et al., 2014
Other VOCs	GC-FID/preconcentration	Miller and Rhoderick, 1995; Rhoderick and Yen, 2006

a GC, gas chromatography; FID, flame ionization detection; CRDS, cavity ring-down spectroscopy; FTIR, fourier transform infrared spectroscopy; ECD, electron capture detection; MS, mass spectrometry.

**Table 2:** Cylinder material and internal treatments studied. DOI: https://doi.org/10.1525/elementa.366.t2

Cylinder metal	Internal treatment	Abbreviation	Specialty gas company	Treatment first available
Aluminum	Aculife IV	Al-Acu-IV	Scott Specialty Gases^a^	1980s
Aluminum	Aculife IV+III	Al-Acu-IV+III	Scott Specialty Gases^a^	1990s
Aluminum	Aculife VIII	Al-Acu-VIII	Scott Specialty Gases^a^	2000s
Aluminum	No treatment	Al	Luxfer Gas Cylinders (UK)	
Aluminum	SpectraShield	Al-SpecShield	Spectra Gases^b^	1990s
Aluminum	Megalife	Al-MegaL	Airgas^a^	2010s
Aluminum	Spectraseal	Al-SS-BOC	BOC, UK	1990s
Aluminum	Experis	Al-Experis	Air Products, Belgium	2000s
Carbon Steel	Nickel plated	CS-Ni	Airgas^a^	2000s
Stainless Steel	Silconert 2000	SS-Silco	Swagelok with SilcoTek	2010s
Aluminum	T-Coat	Al-TCoat	Takachiho, Japan	2010s

a Now Airgas USA, LLC (an Air Liquide Company).

b Now part of Linde.

**Table 3:** Stability data for VOCs in a 30 L Al-Acu-IV cylinder. DOI: https://doi.org/10.1525/elementa.366.t3

VOC	Mole Fraction in nmol mol^−1^ (Uncertainty ± 0.20 nmol mol^−1^)^a^			
	Jan 1989	Aug 1989	Aug 1992	Aug 2002
*bromomethane*	*5.42*	*5.45*	*2.02*	
vinyl chloride	5.23	5.20	5.17	5.29
methylene chloride	5.02	4.91	4.92	4.95
*1,3-butadiene*	*4.74*	*3.44*	*2.46*	
trichlorofluoromethane	5.08	5.10	5.10	5.12
chloroform	4.95	4.94	4.93	5.01
1,2-dichloroethane	5.01	4.94	4.97	
*1,1,1-trichloroethane*	*5.04*	*5.05*	*4.76*	*4.74*
*carbon tetrachloride*	*4.95*	*4.91*	*4.87*	*4.51*
1,2-dichloropropane	5.01	5.07	5.03	4.84
*trichloroethylene*	*5.02*	*5.05*	*5.02*	*4.76*
benzene	5.00	4.97	4.95	
*1,2-dibromoethane*	*4.89*	*4.94*	*4.75*	*1.15*
tetrachloroethylene	4.99	5.04	5.00	4.91
toluene	4.92	4.91	4.91	4.85
chlorobenzene	5.02	5.01	5.00	4.80
*ethylbenzene*	*4.70*	*4.70*	*4.71*	*4.43*
*o-xylene*	*5.08*	*4.94*	*5.11*	*4.66*

a Uncertainty is at the approximate 95% confidence interval.

**Table 4:** Stability data for key VOCs in a 30 L Al-SpecShield cylinder. DOI: https://doi.org/10.1525/elementa.366.t4

VOC	Mole Fraction in nmol mol^−1^, Uncertainty at 95% confidence interval		
	Jan 2004	Feb 2007	Jun 2010
dichlorodifluoromethane	1.01 ± 0.03	1.01 ± 0.02	1.00 ± 0.02
vinyl chloride	0.60 ± 0.01	0.59 ± 0.02	0.60 ± 0.02
*1,3-butadiene*	*1.49 ± 0.12*	*1.32 ± 0.14*	*1.21 ± 0.14*
*bromomethane*	*2.08 ± 0.17*	*1.83 ± 0.20*	*1.65 ± 0.22*
trichlorofluoromethane	1.77 ± 0.06	1.77 ± 0.07	1.76 ± 0.07
dichloromethane	1.84 ± 0.11	1.86 ± 0.11	1.84 ± 0.11
chloroform	0.24 ± 0.01	0.25 ± 0.02	0.24 ± 0.02
1,1,2-trichlorotrifluoroethane	0.53 ± 0.02	0.53 ± 0.02	0.53 ± 0.02
1,1,1-trichloroethane	1.32 ± 0.04	1.33 ± 0.04	1.32 ± 0.04
*carbon tetrachloride*	*0.17 ± 0.01*	*0.08 ± 0.04*	*0.02 ± 0.02*
1,2-dichloroethane	0.45 ± 0.02	0.42 ± 0.02	0.44 ± 0.22
benzene	2.59 ± 0.07	2.59 ± 0.05	2.59 ± 0.05
trichloroethylene	0.44 ± 0.02	0.44 ± 0.02	0.44 ± 0.02
*cis-1,3-dichloropropene*	*2.68 ± 0.03*	*2.21 ± 0.45*	*1.69 ± 0.45*
*trans-1,3-dichloropropene*	*2.68 ± 0.09*	*1.44 ± 0.67*	*0.61 ± 0.67*
toluene	4.38 ± 0.08	4.38 ± 0.15	4.38 ± 0.15
tetrachloroethylene	0.52 ± 0.03	0.52 ± 0.02	0.52 ± 0.02
*1,2-dibromoethane*	*0.95 ± 0.03*	*0.89 ± 0.11*	*0.84 ± 0.11*
chlorobenzene	1.81 ± 0.04	1.80 ± 0.05	1.80 ± 0.05
ethylbenzene	2.77 ± 0.07	2.76 ± 0.06	2.76 ± 0.06
*p*-xylene	2.60 ± 0.06	2.59 ± 0.06	2.59 ± 0.06
*m*-xylene	2.63 ± 0.12	2.61 ± 0.07	2.61 ± 0.07
*o*-xylene	2.70 ± 0.09	2.68 ± 0.06	2.68 ± 0.06
*Styrene*	*3.5 ± 0.2*	*3.1 ± 0.6*	*2.8 ± 0.6*
1,3-dichlorobenzene	2.57 ± 0.09	2.52 ± 0.09	2.57 ± 0.09
1,4-dichlorobenzene	2.71 ± 0.09	2.67 ± 0.19	2.71 ± 0.19
1,2-dichlorobenzene	2.69 ± 0.07	2.59 ± 0.28	2.69 ± 0.28

**Table 5a:** Summarized rates of change of VOCs in treated cylinders (% yr^−1^): non-methane hydrocarbons. DOI: https://doi.org/10.1525/elementa.366.t5a

	Cylinder material and treatment			
	Al-Acu-IV	Al-SpecShield	Al-SS-BOC	Al-Experis
ethane	<±0.2^a,b^		<±0.2^a^	<±0.2^a^
ethene			<±0.2^a^	<±0.2^a^
propane	<±0.2^a,b^		<±0.2^a^	<±0.2^a^
propene	<±0.2^a^; −1.3^b^		<±0.2^a^	<±0.2^a^
*iso*-butane	<±0.2^a,b^		<±0.2^a^	<±0.2^a^
*n*-butane	<±0.2^a,b^		<±0.2^a^	<±0.2^a^
acetylene			−50^a^	<±0.2^a^
*trans*-2-butene			<±0.2^a^	<±0.2^a^
*iso*-butene	<±0.2^a^; −2.4^b^		<±0.2^a^	<±0.2^a^
1-butene			<±0.2^a^	<±0.2^a^
*cis*-2-butene			<±0.2^a^	<±0.2^a^
*iso*-pentane	<±0.2^a,b^		<±0.2^a^	<±0.2^a^
*n*-pentane	<±0.2^a,b^		<±0.2^a^	<±0.2^a^
1,3-butadiene	−13.2^a,b^	−2.9^a^	−1.3^a^	<±0.2^a^
*trans*-2-pentene			<±0.2^a^	<±0.2^a^
1-pentene	<±0.2^a^; −3.4^b^		<±0.2^a^	<±0.2^a^
2-methylpentane			<±0.2^a^	<±0.2^a^
*n*-hexane	<±0.2^a,b^		<±0.2^a^	<±0.2^a^
isoprene	<±0.2^a^		−2.0^a^	<±0.2^a^
*n*-heptane	<±0.2^b^		<±0.2^a^	<±0.2^a^
benzene	<±0.2^a,b^		<±0.2^a^	<±0.2^a^
*iso*-octane	<±0.2^b^		<±0.2^a^	<±0.2^a^
*n*-octane	<±0.2^a,b^		<±0.2^a^	<±0.2^a^
toluene	<±0.2^a,b^	<±0.2^a^	<±0.2^a^	<±0.2^a^
ethylbenzene	−0.4^a^	<±0.2^a^	−2.7^a^	<±0.2^a^
*m*-xylene		<±0.2^a^	−1.3^a^	<±0.2^a^
*p*-xylene		<±0.2^a^	−1.3^a^	<±0.2^a^
*o*-xylene	<±0.2^a,b^; −0.6^a^	<±0.2^a^	<±0.2^a^	<±0.2^a^
1,3,5-trimethylbenzene			<±0.2^a^	<±0.2^a^
1,2,4-trimethylbenzene			<±0.2^a^	<±0.2^a^
1,2,3-trimethylbenzene			<±0.2^a^	<±0.2^a^
nonane	<±0.2^b^			
decane	<±0.2^a,b^			

a Compound in nitrogen at (1 to 10) nmol mol^−1^.

b Compound in nitrogen at 200 pmol mol^−1^.

**Table 5b:** Summarized rates of change of VOCs in treated cylinders (% yr^−1^): monoterpenes, formaldehyde and dimethyl sulfide. DOI: https://doi.org/10.1525/elementa.366.t5b

	Cylinder material and treatment						
	Al-Acu-IV	Al-Acu-IV+VIII	Al-Acu-VIII	Al (no treatment)	Al-SS-BOC	Al-Experis	CS-Ni
*p*-cymene	2.9^a^					<±0.5^b,c^	
*α*-pinene	19^a,c^	<±0.5^a^		−84^a^		<±0.5^b,c^	184^a,c^
*β*-pinene	−81^a^					<±0.5^b^	−68^a^
*R*-limonene	11.5^a^	−53^a^		−126^a^		<±0.5^b,c^	
1,8-cineole	<±0.5^a^	−126^a^		−185^a^		<±0.5^b,c^	−131^a,c^
myrcene	−3.2^a^					<±0.5^b^	
sabinene	−100^a^						
camphene	1000^a,c^					<±0.5^b,c^	
3-carene	<±0.5^a^	−15^a^		−13^a^		<±0.5^b,c^	−161^a,c^
*α*-terpinene						−8.8^b,c^	
formaldehyde		<1.0^d^	−1.2^d^	−0.7^d^	−0.27^d^	−2.5^d^	
dimethyl sulfide		−4%^e,f^		−28%^e,f^		<±1^a^; −6^e^	

a Compound in nitrogen at (1 to 10) nmol mol^−1^.

b Compound in air at 2 nmol mol^−1^.

c In the presence of *β*-pinene in the same mixture.

d Compound in nitrogen at (1 to 10) μmol mol^−1^.

e Compound in nitrogen at 0.5 nmol mol^−1^.

f Immediate loss of VOC upon preparation.

**Table 5c:** Summarized rates of change of VOCs in treated cylinders (% yr^−1^): oxygenated VOCs. DOI: https://doi.org/10.1525/elementa.366.t5c

	Cylinder material and treatment					
	Al-Acu-IV	Al-MegaL	Al-SS-BOC	Al-Experis	SS-Silco	Al-TCoat
methanol	−15%^a,d^; <±1.0	−75%^b,d^	(−3 to −15)%^a,d^; −0.5^a^	−15%^d^; −10^a^	−6^b^	−1^a^
ethanol	−15%^a,d^; <±1.0	−30%^b,d^	(−1 to −8)%^a,d^; −0.5^a^	−2^a^	−6^b^	−0.7^a^
acetone	0.65^c^; −5%^a,d^; <±1.0	<±1^b^	<±0.5^a^	<±1.0^a^	3^b^	−0.7^a^
acetaldehyde					−6^b^	
methacrolein					−3^b^	
methyl ethyl ketone					3^b^	

a Compound in nitrogen at (1 to 10) μmol mol^−1^.

b Compound in nitrogen at 100 nmol mol^−1^.

c Compound in nitrogen at (1 to 10) nmol mol^−1^.

d Immediate loss of VOC upon preparation.

**Table 5d:** Summarized rates of change of VOCs in treated cylinders (% yr^−1^): halocarbons and other VOCs. DOI: https://doi.org/10.1525/elementa.366.t5d

	Cylinder material and treatment	
	Al-Acu-IV	Al-SpecShield
dichlorodifluoromethane	<±0.5^a^	<±0.2^b^
trichlorofluoromethane	<±0.5^a^	<±0.2^b^
1,1,2-trichlorotrifluoroethane	<±0.5^a^	<±0.2^c^
1,1,1,2-tetrfluoroethane	<±0.5^a^	
difluorochloromethane	<±0.5^a^	
1,1-difluoro-1-chloroethane	<±0.5^a^	
bromomethane	−17^b^	−3.2^b^
vinyl chloride	<±0.2^b^	<±0.5^c^
dichloromethane	<±0.2^b^	<±0.2^b^
chloroform	<±0.2^b^	<±0.5^c^
1,2-dichloroethane	<±0.2^b^	<±0.5^c^
1,1,1-trichloroethane	−0.44^b^; <±0.5^d^	<±0.2^b^
carbon tetrachoride	−0.7^b^; −1.4^c^; <±0.5^d^	−14^c^
1,2-dichloropropane	<±0.2^b^	<±0.2^b^
trichloroethylene	−0.38^b^; <±0.5^d^	<±0.5^c^
benzene	<±0.2^b^	<±0.2^b^
1,2-dibromoethane	−5.6^b^; <±0.5^d^	−1.8^b^
tetrachloroethylene	<±0.2^b^	<±0.5^c^
chlorobenzene	<±0.2^b^	<±0.2^b^
*cis*-1,3-dichloropropene		−5.7^b^
*trans*-1,3-dichloropropene		−12^b^
styrene		−3.1^b^
1,2-dichlorobenzene		<±0.2^b^
1,3-dichlorobenzene		<±0.2^b^
1,4-dichlorobenzene		<±0.2^b^

a Compound in air at (20 to 300) pmol mol^−1^

b Compound in nitrogen at (1 to 10) nmol mol^−1^.

c Compound in nitrogen at 200 pmol mol^−1^.

d Compound in air at 200 pmol mol^−1^, contained in pre-1990 alloy cylinder.

## Footnotes

1 There have been no publications by these NMIs relating to data showing instability of VOCs in non-treated cylinders. The authors will not introduce data in this paper to illustrate this known instability.
